# Life Course Predictors of Allostatic Load Among LGBTQ Older Adults

**DOI:** 10.1093/geroni/igaa057.2615

**Published:** 2020-12-16

**Authors:** Charles Hoy-Ellis, Hyun Kim, Karen Fredriksen Goldsen

**Affiliations:** 1 University of Utah, Salt Lake City, Utah, United States; 2 University of Washington, Seattle, Washington, United States

## Abstract

LGBTQ older adults are at significantly increased risk for poor mental and physical health, likely consequential to lifelong bias. Allostatic load (AL), the net effect of “wear and tear” on the body resulting from repeated, chronic over-activation of the psychophysiological stress response system. Utilizing the Health Equity Promotion Model, the aim of this study was to test potential life course predictors of AL, including interpersonal violence, legal marriage, and identity management in a sample of LGBTQ adults 50 to 97 years of age (n=317). Results from a series of hierarchical linear regression models showed that adult physical abuse and late identity disclosure for those who had been in an opposite-sex marriage predicted higher AL in this sample of LGBTQ older adults, indicating need for increased research on bias over the life course as contributory to AL and biopsychosocial dysfunction among LGBTQ older adults.

